# Interactions of problematic mobile phone use and psychopathological symptoms with unintentional injuries: a school-based sample of Chinese adolescents

**DOI:** 10.1186/s12889-016-2776-8

**Published:** 2016-01-28

**Authors:** Shuman Tao, Xiaoyan Wu, Yuhui Wan, Shichen Zhang, Jiahu Hao, Fangbiao Tao

**Affiliations:** 1 Department of Maternal, Child & Adolescent Health, School of Public Health, Anhui Medical University, 81 Meishan Road, Hefei, Anhui Province 230032 China; 2 Anhui Provincial Key Laboratory of Population Health & Aristogenics, Hefei, 230032 China

**Keywords:** Mobile phone, Psychopathological symptoms, Unintentional injuries, Interaction, Adolescents

## Abstract

**Background:**

Unintentional injuries are a major contributor to morbidity and mortality in adolescents. Mobile phone use in certain circumstances (e.g., driving, cycling, walking) and mental health conditions are risk factors for unintentional injury. However, research on the interactions between problematic mobile phone use (PMPU) and psychopathological symptoms in unintentional injuries is limited. The present study aimed to determine the prevalence of unintentional injuries (road traffic injuries, pedestrian collisions, and falls) and examined interactions of PMPU and psychopathological symptoms with unintentional injuries in a school-based sample of Chinese adolescents.

**Methods:**

A total of 14,221 students (6915 middle school students and 7306 high school students) were randomly selected from 32 schools in four cities in China in 2012. The sample comprised 6712 boys and 7509 girls with a mean age of 15.12 years (standard deviation 1.89 years). PMPU, psychopathological symptoms, and unintentional injuries were measured with validated instruments. Chi-square tests and multivariable logistic regression were used to analyze the rates of unintentional injuries, the relationship with PMPU and psychopathological symptoms, and the interactions of PMPU and psychopathological symptoms with unintentional injuries.

**Results:**

The prevalence of road traffic injuries, pedestrian collisions, and falls were 4.9, 16.2, and 10.1 %, respectively. The rates of unintentional injuries were higher among students with PMPU and psychopathological symptoms. Interaction analysis indicated that psychopathological symptoms were associated with a greater increase in the likelihood of unintentional injuries for adolescents with PMPU than for those without PMPU.

**Conclusions:**

The findings indicate that unintentional injuries in adolescents are an important public health issue in China that merit further research. Intervention programs must consider the adolescents’ behavioral and psychological health.

**Electronic supplementary material:**

The online version of this article (doi:10.1186/s12889-016-2776-8) contains supplementary material, which is available to authorized users.

## Background

Mobile phones are now considered an essential part of everyday life, embraced by all age groups and demographics. The Ministry of Industry and Information Technology reported that in 2011 there were nearly 1 billion mobile phone subscribers in China; the number of subscribers increased by more than 81 million to over 940 million in the first 8 months of the year [1]. However, problematic mobile phone use (PMPU) has rapidly increased as a risk behavior worldwide. PMPU is defined as an inability to regulate one’s use of the mobile phone, which eventually involves negative consequences in daily life [2]. In a Korean sample, Lee et al. reported that 16 % of middle school students were “addicted” to their phones [3]. In a Tunisian population, Halayem et al. reported that 26 % of participants suffered from excessive mobile phone use [4]. A study in the United Kingdom found the prevalence of PMPU among 1026 students was 10 %, with the typical problematic users being adolescents aged 11–14 years [5].

In addition, the negative effects of PMPU should not be underestimated. The risk of distraction from mobile phones has grown dramatically over recent years, with this distraction connected to unintentional injuries, such as involvement in road traffic accidents or pedestrian injuries. Road traffic accident rates are higher for adolescent drivers than for any other age group, and driving while distracted might contribute to these high accident rates. A study in the United States found that nearly half of high school students aged ≥16 years reported texting while driving during the past 30 days, with this group more likely to engage in additional risky motor vehicle behaviors [6]. Zhao et al. noted that a greater reported frequency of mobile phone use while driving was likely to increase the overall risk of accident involvement [7]. Mobile phone use while riding a bicycle may also increase the accident risk for cyclists [8]. Furthermore, mobile phones have been found to have a negative effect on perception, potentially forming a threat to cyclist traffic safety [9].

Pedestrian injury and unintentional falls are major public health issues. Stavrinos et al. found that college students frequently talked on the phone while walking, and have increased pedestrian injury rates compared with other age groups. They conducted two experiments, with the results suggesting mobile phone conversations distracted pedestrians to a level that compromised safety [10]. Another study suggested that pedestrian behavior was generally riskier when participants were using mobile Internet and crossing the street than when crossing the street with no distraction. The hypothetical mechanism suggested for pedestrian risk was that the increased cognitive load caused by a distracting device reduced the efficacy of the cognitive process in perceiving relevant environmental conditions at roadside and making safe decisions to initiate motoric processes [11].

The number of mobile phone-related injuries has increased, and more research is required to evaluate the risks caused by mobile phone distraction in all aspects of life [12]. Studies of falls have mainly focused on older adults, and research on falls in children or adolescents is lacking. Globally, almost 50 % of the total number of disability-adjusted life years lost to falls occurred in children under 15 years of age [13]. Falls are gaining attention as a public health issue and may be associated with distraction from mobile phones; therefore, falls were included as an outcome in the present study.

There has also been a growing interest in the potential influence of mental health factors on the risk for unintentional injuries. Poor psychological symptoms include inattention, impulsion, errors in perception and judgment, and psychomotor dysfunction. All of these are potential mechanisms that may explain how poor psychological symptoms increase the risk of unintentional injuries [14]. A cross-sectional study showed that participants with depressive symptoms had a higher prevalence of unintentional injuries [15]. Another study indicated that pre-existing depression was associated with unintentional injury in rural Australians [16]. Furthermore, psychological symptoms were shown to influence young drivers to engage in mobile phone use. Weller et al. investigated if risk perception could predict the tendency of younger drivers to engage in calling while driving, with lower risk perception toward calling while driving associated with higher risk driving behavior [17]. Perceived risk has also been negatively correlated with answering a phone call while driving [18]. It may be that people who use mobile phones while driving are more inclined to ignore the associated injury risks because of their own errors in perceived control. As psychological symptoms influence mobile phone use in driving behavior, they may also increase the risk of mobile phone-related injuries.

Given the evidence that unintentional injuries among adolescents are associated with PMPU and with psychopathological symptoms, the present study aimed to examine (1) associations of PMPU and psychopathological symptoms with self-reported unintentional injuries and (2) whether the interaction of PMPU and psychopathological symptoms increased the likelihood of unintentional injuries among Chinese adolescents.

## Methods

### Study design and participants

The study protocol was approved by the Ethics Committee of Anhui Medical University. Data for the present analysis were collected in 2012. Participants were recruited from public middle and high schools (32 schools in total) from four cities (Shenyang, Xinxiang, Guangzhou, and Chongqing) in China. Students were randomly selected using stratified cluster random sampling in two steps. The first step was the selection of eight schools that met inclusion criteria: located in urban and rural areas; represented different levels of quality of education; included one key middle school, one general middle school, one provincial demonstration high school, and one city demonstration high school from urban areas as well as two general middle schools, one general high school and one city demonstration high school from rural areas. The second step involved randomly selecting three classes in each grade from each school, giving a total of 288 classes. All students in these classes (*n* = 14,665) were invited to participate, and 14,221 provided useable data (response rate =97.0 %). Data collection was completed in classroom settings. Teachers, school administrators, and staff distributed questionnaires to the participating students and instructed them to complete the survey anonymously. Most students took approximately 30–45 min to complete the questionnaire. Written informed consent was obtained from all participants (students and parents) before completing the survey.

### Materials

#### Demographic information

Participants were asked about their city, sex, grade, registered permanent residence, any siblings, perceived family economic status, parental expectations, academic performance, number of friends, and cigarette and alcohol use. To determine registered permanent residence, participants were asked “Where are you from?” Responses were categorized as urban or rural. Siblings were determined by the question: “Are there any siblings in your family?” Responses were recoded as “Yes” or “No.” Perceived family economic status was measured with the question “How well off is your family compared with other families in your region?” Responses were recoded into three categories: low, medium, and high. Academic performance was measured with the question “How do you feel about the level of your academic performance in your class?” Response options were “Low,” “Medium,” and “High.” Participants’ number of friends was measured with the question “How many close friends do you have?” Responses were recoded into four categories: none, 1–2, 3–5, and ≥6. Measurement of cigarette and alcohol use was based on the Young Risk Behavior Surveillance System questionnaire [19]. Cigarette smoking was measured with the question “During the past 30 days, on the days you smoked, how many cigarettes did you smoke per day?” Smoking was defined as smoking at least one cigarette per day. Alcohol drinking was measured with the question “During the past 30 days, on how many days did you have at least one drink of alcohol?” Alcohol drinking was defined as drinking at least once during the past 30 days.

#### PMPU

We assessed PMPU with the Self-rating Questionnaire for Adolescent Problematic Mobile Phone Use (SQAPMPU), which is a standardized questionnaire appropriate for evaluating PMPU in adolescents developed by Tao et al. [20] (Additional file 1). The SQAPMPU comprises 13 items (e.g., “I lose sleep due to the time I spend on my mobile phone,” “I become irritable if I have to switch off my mobile phone for meetings, dinner engagements, or at the movies,” “I feel lost without my mobile phone”) and covers three dimensions including withdrawal symptoms, craving, and physical and mental health status. Responses to each item are on a five-point Likert scale (1 = Not true at all, 2 = Slightly true, 3 = Moderately true, 4 = Strongly true, and 5 = Extremely true). The validity and reliability of the SQAPMPU have been previously examined. Exploratory factor analysis showed the variance cumulative contribution rate was 59.13 %. Confirmatory factor analysis showed that the value of Root Mean Square Error Approximate was 0.067, and the values of fit indices (Norm Fitting index, Relative Fitting Index, Comparative Fitting Index, Goodness of Fit Index, and Adjusted Goodness of Fit Index) were above 0.9. All of the indicators were significant and had good construct validity. To determine the concurrent validity of the SQAPMPU, correlations between the SQAPMPU and the Mobile Phone Problem Use Scale developed by Bianchi and Phillips [21] were investigated in 238 students. The results showed a significant correlation, with a Pearson correlation coefficient of 0.862 (*P* < 0.001).

The Cronbach’s alpha coefficient and split-half reliability were 0.87 and 0.80, respectively. In the present study, the Cronbach’s alpha coefficient was 0.94. The scores ranged between 13 and 65, with the 75th percentile used as the cutoff point, meaning scores were recoded as “No” (<*P*_75_) or “Yes” (**≥** *P*_75_). The distribution of the SQAPMPU scores for the middle schools, high schools and the total sample differed: *P*_75_ was 20 in middle schools, 26 in high schools and 24 in the total sample. As we performed separate analyses in middle schools and high schools, we did not use *P*_75_ of the total sample. Therefore, middle school students with scores ≥20 and high school students with scores ≥26 were defined as problematic mobile phone users.

#### Psychopathological symptoms

The Multidimensional Sub-health Questionnaire of Adolescents (MSQA) is a multidimensional, self-reported symptom inventory developed in China by Tao et al. [22]. The MSQA contains physical and psychological domains and consists of 71 items in total: 32 items for physical symptoms and 39 items for psychological symptoms. We used the 39 psychological symptoms items to evaluate psychopathological symptoms, which were divided into three symptom dimensions: emotional symptoms (17 items), behavioral symptoms (9 items), and social adaptation problems (13 items). Emotional symptoms included depression, anxiety symptoms, and tension. Behavioral symptoms included hostile behaviors, losing control, and inattention. Social adaptation problems included bad relationships with school, family, and friends. Each item has six response options based on the duration of each symptom (none or <1 week, 7 days up to 2 weeks, >2 weeks up to 1 month, >1 month up to 2 months, >2 months up to 3 months, >3 months). In the present analysis, no symptoms and symptom durations of less than 1 month were classified as 0, and symptom durations of 1 month or more were classified as 1. Participants with total scores of ≥8 were defined as having psychopathological symptoms.

#### Unintentional injuries

Unintentional injuries were those that occurred prior to taking part in this study. Our investigation covered three types of unintentional injuries, using three questions: “Have you ever experienced road traffic injuries while using your mobile phone when cycling or driving on a street or the campus?”; “Have you ever experienced a pedestrian collision while using your mobile phone when walking on a street or the campus?” (This referred to colliding with someone or something or being knocked into by other people or hit by something else, such as vehicles, buildings, or trees); and “Have you ever fallen while using your mobile phone when walking on a street or the campus?” (This referred to falling to the ground, floor, or other lower level; falls from animals, burning buildings, and transport vehicles, and falls into fire, water, and machinery were excluded). Responses to these questions were divided into “Yes” and “No” categories. If “yes,” participants were asked to indicate how frequently the injuries happened: occasionally (1–2 times) and frequently (at least 3 times). As the answer “frequently” was rarely chosen, we only used a binary (yes/no) answer for the analysis.

### Statistical analysis

Bivariate analysis (chi-square tests for categorical variables) was used to assess the relationship between participants’ demographic factors and PMPU, psychopathological symptoms, and unintentional injuries. Multivariate logistic regression was used to examine the associations of PMPU and psychopathological symptoms with unintentional injuries, and to assess the interaction of PMPU and psychopathological symptoms with unintentional injuries. Statistical significance was set at *P* < 0.05 (two-tailed). Statistical analyses were conducted with SPSS software version 10.0 (SPSS Inc, Chicago, IL, USA).

## Results

### Characteristics of the study sample

Table 1 presents the characteristics of the sample stratified by the type of school. Overall, 14,221 students participated in the assessments. Participants from middle schools (*n* = 6915) accounted for 48.6 % of the total sample. The mean age was 15.1 years (standard deviation [SD] = 1.9 years); 6712 were boys (47.2 %) and 7509 were girls (52.8 %). There were differences in PMPU and psychopathological symptoms between the four cities. There were no sex-based differences in PMPU for high schools, but in middle schools, PMPU was more common in girls (27.4 % of girls vs. 24.9 % of boys, *P* < 0.05). Psychopathological symptoms were more common in both middle school and high school girls (middle school: 15.7 % of girls vs. 13.3 % of boys; high school: 28.7 % of girls vs. 25.3 % of boys; *P* < 0.05). PMPU was lower in middle school students from rural areas (23.9 % vs. 28.0 %; *P* < 0.001). PMPU was also significantly lower in students with siblings (23.4 % vs. 28.5 %, *P* < 0.01). Students in middle schools who reported low perceived family economic status showed a higher proportion of PMPU and psychopathological symptoms. A higher rate of psychopathological symptoms was also observed in high school students who reported a low perceived family economic status. There were significant differences in PMPU and psychopathological symptoms between different levels of parental expectations or self-reported academic performance; the rates of PMPU and psychopathological symptoms were highest in participants with parents with low expectations or low self-reported academic performance. Students in middle school who reported they had fewer close friends showed a higher proportion of PMPU and psychopathological symptoms, with the highest rate of psychopathological symptoms in high school students who reported no close friends. Students who were current smokers and alcohol drinkers had a higher percentage of PMPU and psychopathological symptoms in both school types.

**Table 1:** Characteristics of the 2012 sample of Chinese adolescents (*n* = 14,221)

Variables	Middle school				High school			
	PMPU		Psychopathological symptoms		PMPU		Psychopathological symptoms	
	n (%)	*χ* ^*2*^	n (%)	*χ* ^*2*^	n (%)	*χ* ^*2*^	n (%)	*χ* ^*2*^
Area								
Shenyang	407 (26.5)		231 (15.1)		650 (33.9)		645 (33.6)	
Xinxiang	343 (15.6)	223.37^**^	224 (10.2)	59.92^**^	452 (22.8)	149.14^**^	494 (24.9)	56.06^**^
Guangzhou	575 (30.7)		290 (15.5)		281 (19.2)		348 (23.8)	
Chongqing	486 (36.8)		256 (19.4)		659 (33.9)		499 (25.7)	
Gender								
Boys	863 (24.9)	5.57^*^	459 (13.3)	8.19^*^	946 (29.1)	3.78	823 (25.3)	10.42^*^
Girls	948 (27.4)		542 (15.7)		1096 (27.0)		1163 (28.7)	
Registered permanent residence								
Rural	733 (23.9)	15.17^**^	418 (13.6)	3.26	1000 (27.9)	0.006	1009 (28.2)	3.40
Urban	1078 (28.0)		583 (15.2)		1042 (28.0)		977 (26.2)	
Any siblings								
Yes	733 (23.4)	23.40^**^	430 (13.7)	2.67	931 (26.1)	11.44^*^	976 (27.4)	0.15
No	1078 (28.5)		571 (15.1)		1111 (29.7)		1010 (27.0)	
Perceived family economic								
Low	188 (31.6)		135 (22.7)		351 (27.3)		465 (36.1)	
Medium	1220 (25.2)	12.09^*^	676 (14.0)	36.71^**^	1462 (27.7)	3.13	1294 (24.5)	74.47^**^
High	403 (27.2)		190 (12.8)		229 (30.7)		227 (30.4)	
Parental expectations								
Low	46 (42.2)		38 (34.9)		31 (44.9)		39 (56.5)	
Medium	445 (28.2)	19.99^**^	254 (16.1)	43.35^**^	580 (28.2)	10.23^*^	508 (24.7)	37.28^**^
High	1320 (25.3)		709 (13.6)		1431 (27.6)		1439 (27.8)	
Academic performance								
Low	546 (35.9)		364 (23.9)		726 (32.7)		805 (36.2)	
Medium	963 (24.3)	99.38^**^	476 (12.0)	140.53^**^	1101 (26.2)	36.18^**^	972 (23.1)	131.79^**^
High	302 (21.2)		161 (11.3)		215 (24.5)		209 (23.8)	
Number of friends								
0	72 (34.8)		84 (40.6)		62 (25.4)		127 (52.0)	
1–2	410 (29.8)	26.36^**^	307 (22.3)	226.74^**^	426 (27.4)	2.56	534 (34.4)	148.50^**^
3–5	693 (26.3)		331 (12.6)		933 (28.8)		814 (25.1)	
≥6	636 (23.6)		279 (10.4)		621 (27.4)		511 (22.5)	
Cigarette use								
Yes	203 (51.9)	141.92^**^	138 (35.3)	145.08^**^	387 (42.3)	106.89^**^	349 (38.1)	63.47^**^
No	1608 (24.6)		863 (13.2)		1655 (25.9)		1637 (25.6)	
Alcohol Use								
Yes	1220 (34.7)	269.32^**^	677 (19.3)	132.64^**^	1582 (31.3)	91.50^**^	1481 (29.3)	37.24^**^
No	591 (17.4)		324 (9.5)		460 (20.4)		505 (22.4)	

PMPU refers to problematic mobile phone use, this applies to all tables that use the abbreviation

^*^refers to *P* < 0.05; ^**^ refers to *P* < 0.001

### Prevalence of unintentional injuries and self-reported PMPU and psychopathological symptoms

We examined the associations of PMPU and psychopathological symptoms with unintentional injuries stratified by school (Table 2). Self-reported PMPU and psychopathological symptoms were significantly associated with unintentional injuries (traffic accidents, pedestrian collisions, and accidental falls) in both middle and high schools. Participants who reported PMPU and psychopathological symptoms were more likely to experience unintentional injuries (Table 3). A chi-square test indicated that the rates of unintentional injuries were significantly higher for students with PMPU than for non-PMPU from middle school (traffic injuries 9.3 % vs. 2.2 %, pedestrian collisions 28.4 % vs. 7.6 %, falls 19.9 % vs. 4.7 %; *P* < 0.001) and high school (traffic injuries 11.7 % vs. 3.5 %, pedestrian collisions 34.3 % vs. 13.3 %, falls 21.8 % vs. 7.5 %; *P* < 0.001) and significantly higher in students with psychopathological symptoms than those without psychopathological symptoms from middle school (traffic injuries 8.2 % vs. 3.4 %, pedestrian collisions 24.1 % vs. 11.2 %, falls 16.7 % vs. 7.4 %; *P* < 0.001) and high school (traffic injuries 9.4 % vs. pedestrian collisions 4.4 %, 27.7 % vs. 16.0 %, falls 17.6 % vs. 9.2 %; *P* < 0.001).

**Table 2:** Prevalence of unintentional injuries with reported problematic mobile phone use and psychopathological symptoms in Chinese adolescents in 2012, stratified by school

Variables	Road traffic injuries		Pedestrian collision		Falls	
	n (%, 95 % *CI*)	*χ* ^*2*^	n (%, 95 % *CI*)	*χ* ^*2*^	n (%, 95 % *CI*)	*χ* ^*2*^
Middle school						
PMPU						
Yes	169 (9.3, 8.0–10.7)	174.68^**^	514 (28.4, 26.3–30.5)	508.89^**^	361 (19.9, 18.1–21.8)	387.60^**^
No	112 (2.2, 1.8–2.6)		388 (7.6, 6.9–8.3)		242 (4.7, 4.2–5.3)	
Psychopathological symptoms						
Yes	82 (8.2, 6.5–9.9)	51.16^**^	241 (24.1, 21.4–26.7)	125.58^**^	167 (16.7, 14.4–19.0)	93.24^**^
No	199 (3.4, 2.9–3.8)		661 (11.2, 10.4–12.0)		436 (7.4, 6.7–8.0)	
High school						
PMPU						
Yes	239 (11.7, 10.3–13.1)	184.26^**^	701 (34.3, 32.3–36.4)	420.87^**^	446 (21.8, 20.0–23.6)	298.01^**^
No	182 (3.5, 3.0–4.0)		699 (13.3, 12.4–14.2)		394 (7.5, 6.8–8.2)	
Psychopathological symptoms						
Yes	187 (9.4, 8.1–10.7)	67.04^**^	551 (27.7, 25.8–29.7)	129.67^**^	350 (17.6, 15.9–19.3)	100.59^**^
No	234 (4.4, 3.8–4.9)		849 (16.0, 15.0–16.9)		490 (9.2, 8.4–10.0)	

^**^refers to *P* < 0.001

**Table 3:** Regression analysis of the independent association of problematic mobile phone use and psychopathological symptoms with unintentional injuries in Chinese adolescents in 2012

Variables	Road traffic injuries		Pedestrian collision		Falls	
	Crude *OR* (95 % *CI*)	Adjusted^a^ *OR* (95 % *CI*)	Crude *OR* (95 % *CI*)	Adjusted^a^ *OR* (95 % *CI*)	Crude *OR* (95 % *CI*)	Adjusted^a^ *OR* (95 % *CI*)
Middle school						
PMPU						
Yes	4.12 (3.20–5.31)^b^	3.93 (3.01–5.12)^b^	4.38 (3.77–5.08)^b^	3.56 (3.05–4.15)^b^	4.55 (3.81–5.44)^b^	3.91 (3.25–4.71)^b^
No	Ref.	Ref.	Ref.	Ref.	Ref.	Ref.
Psychopathological symptoms						
Yes	1.70 (1.29–2.25)^b^	1.43 (1.07–1.92)^c^	1.69 (1.42–2.02)^b^	1.44 (1.20–1.73)^b^	1.65 (1.35–2.02)^b^	1.38 (1.12–1.71)^b^
No	Ref.	Ref.	Ref.	Ref.	Ref.	Ref.
High school						
PMPU						
Yes	3.27 (2.66–4.02)^b^	3.23 (2.61–4.00)^b^	3.10 (2.74–3.51)^b^	2.81 (2.47–3.19)^b^	3.10 (2.67–3.61)^b^	2.91 (2.49–3.40)^b^
No	Ref.	Ref.	Ref.	Ref.	Ref.	Ref.
Psychopathological symptoms						
Yes	1.69 (1.37–2.08)^b^	1.62 (1.30–2.01)^b^	1.57 (1.38–1.78)^b^	1.54 (1.34–1.76)^b^	1.61 (1.38–1.89)^b^	1.57 (1.34–1.85)^b^
No	Ref.	Ref.	Ref.	Ref.	Ref.	Ref.

^a^Adjusted for area, gender, registered permanent residence, any siblings, perceived family economic, parental expectations, academic performance, number of friends, cigarette use and alcohol use

^b^refers to *P* < 0.001

^c^refers to *P* < 0.05

### Independent association of PMPU and psychopathological symptoms with unintentional injuries

There was a positive association between PMPU and psychopathological symptoms and unintentional injuries (Table 3). In univariate logistic regression analyses, both PMPU and psychopathological symptoms remained independently associated with unintentional injuries. In the models adjusted for area, sex, registered permanent residence, any siblings, perceived family economic status, parental expectations, academic performance, number of friends, cigarette use, and alcohol use, PMPU in middle school students was associated with road traffic injuries (odds ratio [OR] = 3.93, 95 % confidence interval [CI]: 3.01–5.12), pedestrian collisions (OR = 3.56, 95 % CI: 3.05–4.15), and falls (OR = 3.91, 95 % CI: 3.25–4.71). In addition, middle school students with psychopathological symptoms showed positive correlations with road traffic injuries (OR = 1.43, 95 % CI: 1.07–1.92), pedestrian collisions (OR = 1.44, 95 % CI: 1.20–1.73), and falls (OR = 1.38, 95 % CI: 1.12–1.71). We found similar results for high school students, with PMPU being associated with road traffic injuries (OR = 3.23, 95 % CI: 2.61–4.00); pedestrian collisions (OR = 2.81, 95 % CI: 2.47–3.19); and falls (OR = 2.91, 95 % CI: 2.49–3.40). There were significant positive associations between psychopathological symptoms and road traffic injuries (OR = 1.62, 95 % CI: 1.30–2.01), pedestrian collisions (OR = 1.54, 95 % CI: 1.34–1.76), and falls (OR = 1.57, 95 % CI: 1.34–1.85) in high school students.

### Interactions of PMPU and psychopathological symptoms with unintentional injuries

In the second regression analyses, we examined the interactions of PMPU and psychopathological symptoms with unintentional injuries among Chinese adolescents (Table 4). There was a significant additive interaction between PMPU and psychopathological symptoms (*P* < 0.001). Table 4 presents the crude and adjusted OR (95 % CI) for each group in comparison with the reference group (no PMPU/no psychopathological symptoms) for road traffic injuries, pedestrian collisions, and falls. After adjusting for area, sex, registered permanent residence, any siblings, perceived family economic status, parental expectations, academic performance, number of friends, cigarette use, and alcohol use, adolescents in both middle and high school with PMPU and psychopathological symptoms were more likely to experience unintentional injuries. For middle school students, the OR (95 % CI) for road traffic injuries was 5.65 (3.93–8.13), for pedestrian collisions was 5.27 (4.20–6.62), and for falls was 5.13 (3.92–6.71). For high school students, these were: road traffic accidents 5.17 (3.90–6.85), pedestrian collisions 4.37 (3.69–5.19), and falls 4.50 (3.68–5.52).

**Table 4:** Interaction of problematic mobile phone use and psychopathological symptoms on unintentional injuries in Chinese adolescents in 2012

Unintentional injuries	Psychopathological symptoms	Non–PMPU		PMPU	
		Crude *OR* (95 % *CI*)	Adjusted^a^ *OR* (95 % *CI*)	Crude *OR* (95 % *CI*)	Adjusted^a^ *OR* (95 % *CI*)
Middle school					
Road traffic injuries	Yes	1.69 (1.00–2.85)	1.37 (0.80–2.35)	7.02 (5.04–9.77)^b^	5.65 (3.93–8.13)^b^
	No	Ref.	Ref.	4.11 (3.09–5.47)^b^	3.87 (2.87–5.22)^b^
Pedestrian collision	Yes	1.56 (1.15–2.12)^c^	1.27 (0.93–1.73)	7.53 (6.09–9.30)^b^	5.27 (4.20–6.62)^b^
	No	Ref.	Ref.	4.27 (3.62–5.05)^b^	3.41 (2.87–4.06)^b^
Falls	Yes	2.30 (1.65–3.22)^b^	1.87 (1.33–2.64)^b^	7.09 (5.53–9.10)^b^	5.13 (3.92–6.71)^b^
	No	Ref.	Ref.	5.07 (4.15–6.19)^b^	4.33 (3.52–5.33)^b^
High school					
Road traffic injuries	Yes	2.41 (1.78–3.28)^b^	2.26 (1.65–3.09)^b^	5.48 (4.19–7.17)^b^	5.17 (3.90–6.85)^b^
	No	Ref.	Ref.	4.22 (3.23–5.50)^b^	4.10 (3.13–5.37)^b^
Pedestrian collision	Yes	1.50 (1.25–1.80)^b^	1.46 (1.21–1.77)^b^	4.91 (4.17–5.78)^b^	4.37 (3.69–5.19)^b^
	No	Ref.	Ref.	3.00 (2.56–3.50)^b^	2.70 (2.30–3.18)^b^
Falls	Yes	1.82 (1.45–2.28)^b^	1.75 (1.39–2.20)^b^	4.92 (4.05–5.97)^b^	4.50 (3.68–5.52)^b^
	No	Ref.	Ref.	3.38 (2.79–4.10)^b^	3.14 (2.58–3.82)^b^

^a^Adjusted for area, gender, registered permanent residence, any siblings, perceived family economic, parental expectations, academic performance, number of friends, cigarette use and alcohol use

^b^refers to *P* < 0.001

^c^refers to *P* < 0.05

## Discussion

Unintentional injuries are a public health challenge in China. We reported factors influencing unintentional injuries in a school-based sample of Chinese adolescents. Our findings add new data contributing to the understanding of the role of PMPU and its interaction with psychopathological symptoms in influencing the likelihood of adolescent unintentional injuries. In middle schools, the rate of PMPU was 26.2 % and that of psychopathological symptoms was 14.5 %. In high schools, the rate of PMPU was 27.9 % and that of psychopathological symptoms was 27.2 %. Girls reported a higher rate of PMPU than boys, which is consistent with a study conducted in Southern Taiwan [23]. Girls also indicated greater psychopathological symptoms than boys, which is consistent with another Chinese study [24]. Students from urban areas, without siblings, and who reported a low perceived family economic status were more likely to be classified as PMPU. This might be related to economic development status, family structure, parental care-giving, or other social factors. Students who reported low perceived family economic status, low parental expectations, low academic performance, and fewer close friends reported higher rates of PMPU and psychopathological symptoms. Poor economic conditions has been previously associated with more psychological symptoms of depression [24]. Low parental expectations or academic performance might reduce adolescents’ confidence and motivation. Those with no friends might feel lonely, and loneliness might predict overuse of mobile phones (e.g., time spent during the week exchanging text messages) [25]. Students who reported cigarette and alcohol use were more likely to experience PMPU, a finding consistent with the results in an Iran sample [26].

Findings from our analysis indicated that overall, 4.9 % of students reported road traffic injuries, 16.2 % reported injuries from pedestrian collisions, and 10.1 % reported falls. The rate of road traffic injuries was lower than rates reported in young people in Vietnam in 2004 (14.1 %) and 2009 (10.6 %) [27], and was also lower than that reported for adolescents aged 10–19 years (17.4 %) in Victoria, Australia from 2004–2008 [28]. A study conducted in 2000–2009 in England reported a pedestrian injury rate of 12 %, which was lower than that in our study [29]. Another study found that 23 % (357/1557) of participants reported pedestrian injuries in the past 3 months, which was higher than that in our study [30]. The incidence rate of falls in the present study was similar to that reported among high school students in Lijin County, China (9.8 %) [31].

Our findings supported the hypothesis that PMPU and psychopathological symptoms were associated with unintentional injuries in Chinese adolescents. Compared with non-problematic mobile phone users, those with PMPU experienced significantly more unintentional injuries and there was a significant positive association between PMPU and unintentional injuries. These findings suggest that PMPU might be common in students who drive, cycle, or walk while using mobile phone. Vehicle accident risk has been strongly associated with heightened anticipation about incoming phone calls or messages [32]. Driving tasks involving texting and have been found to be associated with significant impairment in driving performance [33]. Texting, calling, or playing a game on a touch screen mobile phone impacted on cycling performance and increased the risk of collision with vehicles or other traffic participants [34]. Similarly, mobile phones may change the way we walk, and the use of mobile phones by pedestrians causes increased cognitive distraction, reduces awareness of the situation, and increases unsafe behaviors [35]. Pedestrians are less likely to successfully cross the road when conversing on a mobile phone or listening to music [36]. Data from the US Consumer Product Safety Commission on injuries in hospital emergency rooms from 2004–2010 found that mobile phone-related injuries among pedestrians increased relative to total pedestrian injuries; using a mobile phone while walking put pedestrians at risk of accident, injury, or death [37].

The associations between psychological health and the incidence of injury have been previously determined. Chen et al. found that mood and anxiety disorders were significantly associated with fall injuries and that people with anxiety or mood disorders had an increased incidence of fall injuries in all age groups [38]. Another study found that injured adolescents had higher average raw scores of all subscales of the Symptom Check List–90–Revised, and symptoms of anxiety, depression, obsessive-compulsiveness, and somatization were associated with an increased risk of nonfatal unintentional injury in middle and high school students [39]. A poor psychopathology status was associated with an increased risk of unintentional injuries among adolescents, with baseline psychological sub-healthy status being a predictive factor for higher risk of unintentional injuries [40]. Lack of personal control has been associated with injury incidence in university students in 26 countries [41]. The results of these studies are consistent with our findings and support our initial hypothesis that emotional and behavioral symptoms were related to injuries.

Our multivariable logistic regression analyses indicated that students with PMPU were likely to experience unintentional injuries, and the association was enhanced by psychopathological symptoms. PMPU was associated with an increased risk of unintentional injuries among students with psychopathological symptoms compared with students without psychopathological symptoms. A systematic review showed that the frequency of mobile phone use while driving and the psychological effects influenced young drivers’ tendencies to engage in mobile phone use while driving [42]. Therefore, the role of psychological health should be noted in Chinese adolescents, and effective school-based educational programs should encourage students not to be dependent on their mobile phones, promote mental health, and educate students to understand the importance safety.

There are several limitations of our study. First, we used a cross-sectional design, and as the timing of injuries was not examined, causal relationships were not defined. Further studies should consider a prospective cohort design to achieve improvement in study design. Second, PMPU was determined by the SQAPMPU, and the results might differ if other criteria were used. Third, we used self-reported data, and information bias might be inevitable. Despite these limitations, the large sample size in our study provided data that furthers understanding of the significance of psychopathological symptoms as well as the interactions of PMPU with unintentional injuries. It is important that public health policy in China includes planned intervention programs to reduce the rates of unintentional injuries.

## Conclusions

As a public health problem, unintentional injuries in adolescents merit discussion. The findings of our study indicate that PMPU and psychopathological symptoms and their interaction are related to unintentional injuries. Therefore, adolescents’ behavioral and psychological health should be considered in the implementation of effective intervention programs related to mobile phone user habits.

## Additional file

Additional file 1:
**Self-rating Questionnaire for Adolescent Problematic Mobile Phone Use (SQAPMPU).** (DOC 44 kb)

## Abbreviations

CI: Confidence interval
MSQA: Multidimensional sub-health questionnaire of adolescents
OR: Odds ratio
PMPU: Problematic mobile phone use
SQAPMPU: Self-rating questionnaire for adolescent problematic mobile phone use
